# *In Vitro* Antioxidant and Xanthine Oxidase Inhibitory Activities of Methanolic *Swietenia mahagoni* Seed Extracts

**DOI:** 10.3390/molecules14114476

**Published:** 2009-11-06

**Authors:** Geethaa Sahgal, Surash Ramanathan, Sreenivasan Sasidharan, Mohd Nizam Mordi, Sabariah Ismail, Sharif Mahsufi Mansor

**Affiliations:** 1 Centre for Drug Research, Universiti Sains Malaysia, 11800 Penang, Malaysia; 2 Institute for Research in Molecular Medicine, Universiti Sains Malaysia, 11800 Penang, Malaysia

**Keywords:** *Swietenia mahagoni* seed, antioxidant activity, HPTLC

## Abstract

This study examines the *in vitro* antioxidant activities of the methanol extract of *Swietenia mahagoni* seeds (SMCM seed extract). The extract was screened for possible antioxidant activities by free radical scavenging activity (DPPH), xanthine oxidase inhibition (XOI), hydrogen peroxide scavenging activity (HPSA) and ferric-reducing antioxidant power (FRAP) assays. The total phenolic and flavonoid contents were also determined. The extract exhibits antioxidant activity of 23.29% with an IC_50_ value of 2.3 mg/mL in the DPPH radical scavenging method, 47.2% in the XOI assay, 49.5% by the HPSA method, and 0.728 mmol/Fe(II)g in the FRAP method at the concentration tested. The amount of total phenolics and flavonoid contents was 70.83 mg gallic acid equivalent (GAE) and 2.5 ± 0.15 mg of catechin equivalent per gram of dry extract, respectively. High Performance Thin Layer Chromatography (HPTLC) screening indicates the presence of phenolic compounds in the SMCM seed extract. The results indicate that the extract has both high free radical scavenging and xanthine oxidase inhibition activity. The antioxidant activity of SMCM seed extract is comparable with that of other Malaysian tropical fruits and herbal plants.

## 1. Introduction

*Swietenia mahagoni* (Linn.) Jacq. (Meliaceae) is a large, deciduous, and economically important timber tree native to the West Indies. This tree is mainly cultivated at tropical zones, such as India, Malaysia, and Southern China [1]. It is a valuable species closely related to the African genus Khaya and the source of one of the most popular traditional medicines in Africa. The fruit is a brown, egg- to pear-shaped capsule about 6 to 10 cm long. Large trees may produce over 100 capsules, but year to year seed production is irregular. When the fruit is fully ripe, the woody shell splits into five sections from the base upward and falls off to release the seeds. The winged seeds (samaras) are 5 to 6 cm long and turn reddish brown in colour. A capsule may contain up to 60 seeds. The decoction of the bark of these mahoganies is extensively used as febrifuge, which could be associated with its use as an anti-malarial drug. *Swietenia* *mahagoni* seeds have been used as folk medicine for the treatment of hypertension, diabetes, and malaria [2], and they have also been reported to have medicinal value for treatment of cancer, amoebiasis, coughs, chest pains and intestinal parasitism. The biologically active ingredients, tetranortriterpenoids and fatty acids, are considered to be responsible for these therapeutic effects [3].

The role of free radicals in many ailments has been well established. Several biochemical reactions in our body system generate reactive oxygen species, which, if not effectively scavenged by cellular constituents, may lead to various disease conditions [4]. Much research into free radicals has confirmed that foods or plants rich in antioxidants play an essential role in the prevention of free radical related diseases [5,6]. A wide range of antioxidants of synthetic origin such as butylated hydroxytoluene (BHT) has been proposed for use in the treatment of various free radicals related diseases [7,8], but it has been proven that these compounds also show toxic effects like liver damage and mutagenesis [9]. Hence, nowadays the search for natural antioxidants source is gaining much importance. The high antioxidant potential observed in many tropical plants is obviously part of their natural defence mechanism against noxious events causing oxidant damage, e.g. microbial infections. Although, *S. mahagoni* is reported for different folk medical use, the present work was carried out to explore the *in vitro* antioxidant potential of this plant.

## 2. Results and Discussion

### 2.1. Inhibition of Xanthine Oxidase

Xanthine oxidase is a flavoprotein that catalyses the oxidation of hypoxanthine to xanthine and generates superoxide and uric acid [10]. It has been shown that xanthine oxidase inhibitors may be useful for the treatment of hepatic disease and gout, which is caused by the generation of uric acid and superoxide anion radical [11]. SMCM seed methanol extract exhibits anti-superoxide activity. A methanol extract was used in this study due to its polarity and because it was anticipated that it would more effectively extract the polyphenols which possessed antioxidant activity from plant samples. At concentrations of 1 mg/mL the extract inhibited superoxide formation with a value of 47.2 ± 0.005%. The SMCM seed extract showed a lesser antioxidant activity compare to allopurinol (87.51 ± 0.001%). The preventive antioxidant activity of SMCM seed extracts largely comes from their ability to inhibit various oxidative enzymes. However, their role as antioxidants in the human body when orally ingested is unknown, but there are several possibilities. The relevance of the *in vitro* experiments in simplified systems to *in vivo* protection from oxidative damage should be carefully considered. The results obtained from this study indicate that the further *in vivo* evaluation is needed.

**Table 1:** The xanthine oxidased inhibitory activity, hydrogen peroxide scavenging and ferric-reducing antioxidant power (FRAP) activity assays for *Swietenia mahagoni* seed extract.

Assays	Inhibition %, (1 mg/mL)
Inhibition of xanthine oxidase	
SMCM^a^ seed extract	47.21 ± 0.005
Allopurinol	87.51 ± 0.001
Hydrogen peroxide scavenging activity	
SMCM^a^ seed extract	49.51 ± 0.025
Ascorbic acid	51.13 ± 0.010
Ferric-reducing antioxidant power (FRAP)	
SMCM^a^ seed extract	0.728 ± 0.031 mmol Fe (II)/g
Ascorbic acid	0.405 ± 0.048 mmol Fe (II)/g

^a^ SMCM – *Swietenia mahagoni* crude methanolic.

### 2.2. Determination of Hydrogen Peroxide Scavenging Activity

The hydrogen peroxide scavenging activity of SMCM seed extract is given in Table 1. The scavenging ability of this extract is comparable to that of ascorbic acid (extract: 49.5 ± 0.02% and ascorbic acid: 51.1 ± 0.01%). Addition of H_2_O_2_ to cells in cultures can lead to transition metal ion-dependent OH^√^ mediated oxidative DNA damage. Levels of H_2_O_2_ at or below about 20–50 mg seem to have limited cytotoxicity to many cell types [12]. Since phenolic compounds present in the plant extract are good electron donors, they may accelerate the conversion of H_2_O_2_ to H_2_O [13], hence, SMCM seed extracts might also help accelerate the conversion of H_2_O_2_ to H_2_O. 

### 2.3. Ferric-Reducing Antioxidant Power Assay (FRAP)

The FRAP assay is versatile and can be readily applied to methanol extracts of different plants. In this assay, the antioxidant activity is determined on the basis of the ability to reduce ferric (III) iron to ferrous (II) iron. The standard curve was generated in the range of 0.5 to 10 mg/mL of ferrous sulphate and the results were expressed as mmol ferrous ion equivalent per gram of sample dry weight (y = 0.0032x + 0.0897, r^2 ^= 0.969). Table 1 show the results for the ferric-reducing antioxidant power assay. The SMCM seed extract (0.728 ± 0.031 mmol Fe (II)/g) shows approximately two-fold higher ferric reducing capacity compared to the standard reference ascorbic acid (0.405 ± 0.048 mmol Fe (II)/g). Our results also show that the SMCM seed extract has better ferric reducing power than date palm (soft dates, Jiroft: 13.32 ± 0.83; semi dry dates, sahroon: 26.93 ± 1.96 and dry dates, Kharak: 387.34 ± 1.94 µmol Fe (II)/g) from Iran [14].

### 2.4. 2,2-Diphenyl-2-picrylhydrazyl (DPPH) Assay

The scavenging the stable DPPH radical is a widely used method to evaluate the free radical scavenging ability of various samples, including plant extracts [15]. The measured DPPH radical scavenging activity is shown in Table 2. The SMCM seed extract scavenging antioxidant activity was 23.29 ± 0.04% at 1 mg/mL. The scavenging effect on the DPPH radical decreased in the order: ascorbic acid > vitamin E > BHT > SMCM seed extract. The effect of antioxidants on DPPH is thought to be due to their hydrogen donating ability [16]. Although the DPPH radical scavenging abilities of the extracts were significantly lower than those of ascorbic acid, vitamin E and BHT, it was evident that the extracts did show some proton-donating ability and could serve as free radical inhibitors or scavengers, acting possibly as primary antioxidants. The quality of the antioxidants in the extracts was determined by the IC_50_ values shown in Table 2. A low EC_50_ value indicates strong antioxidant activity in a sample. The IC_50_ values of SMCM seed extract was 2.2 mg/mL. Although the IC_50_ values of SMCM seed extract were greater than the reference antioxidant, it was comparable to other tropical fruits which are considered to have a good antioxidant powers (guava IC_50_: 2.1 ± 0.63; star fruit IC_50_: 3.8 ± 2.1, papaya IC_50_: 3.5 ± 0.9 mg/mL and pumpkin seed IC_50_: 5.13 mg/mL) [17,18]. However the SMCM seed extract’s Antioxidant Activity Index (AAI) was only 0.2, suggesting poor antioxidant activity (AAI < 0.5) [19]. Lack of hydrogen donor bioactive constituents in the extract, slow rate of the reaction between DPPH and the substrate molecules and/or reaction of DPPH with eugenol resulting in low readings for antioxidant activity probably might explain the low DPPH antioxidant activity of the SMCM seed extract [20,17,21]. 

**Table 2:** DPPH antioxidant activity of 1 mg/mL *Swietenia mahagoni* (SMCM) seed extract.

(DPPH) radical scavenging Assay	Scavenging Activity
SMCM seed extract	23.29 ± 0.04%
Butylated hydroxytoluene (BHT)	81.04 ± 0.19 %
Ascorbic acid	86.04 ± 0.00 %
Vitamin E	82.41 ± 0.19 %
IC_50 _of SMCM seed extract	2.27 mg/ml
Antioxidant Activity Index (AAI)	0.02

### 2.5. Determination of Phenolic and Flavonoids Contents

Total phenol compounds, as determined by the Folin Ciocalteu method, is reported as gallic acid equivalents by reference to standard curve (y = 9.892x + 0.092 and r² = 0.997). The total phenolic content extract was 26.9 ± 0.26 mg GAE/g of extract. The total flavonoid content was 2.5 ± 0.15 mg of catechin equivalent per gram of sample, respectively, by reference to standard curve (y = 0.0038x + 0.0272 and r² = 0.9985). The SMCM seed extract had good total phenolic content that may be responsible for the antioxidative activities of this extract. Phenols and polyphenolic compounds, such as flavonoids, are widely found in food products derived from plant sources, and they have been shown to possess significant antioxidant activities (22).

**Table 3:** Total phenolic and flavonoid content of 1 mg/mL *Swietenia mahagoni* seed extract.

Assays	SMCM seed extract
Total phenolic content	26.94 ± 0.26^ a^
Total flavonoid content	2.52 ± 0.15^ b^

^a^ Gallic acid equivalents mg/g; ^b ^Catechin equivalents mg/g

### 2.6. HPTLC Bioautography Analysis of Phenolic and Antioxidant Substance

**Figure 1:**
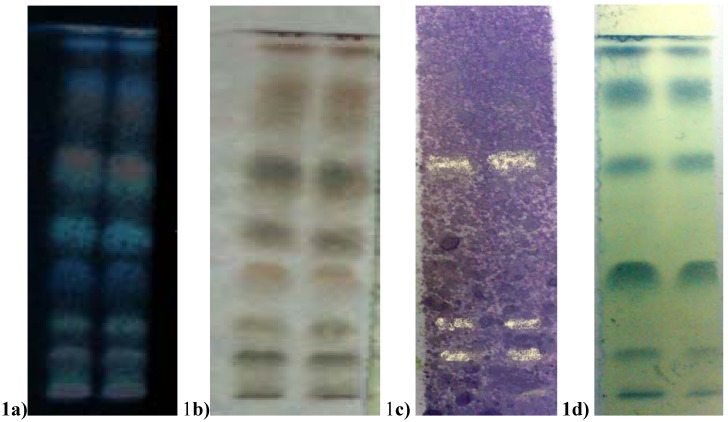
The phenol and antioxidant substance of *Swietenia mahagoni* (SMCM) seed extract in developed TLC (dichloromethane/ethyl acetate 5:1). Figure 1a shows the separated substance at 366 nm and Figure 1b exhibit the coloured substances under normal visible light after treatment with methanol/sulfuric acid (90:10) spray. Figure 1c shows the pale-yellow colour substance on purple colour background for antioxidant activity after spraying with 0.2% DPPH reagent. Figure 1d displays the blue colour of phenolic substances on a yellow background after Folin-Ciocalteu reagent spray.

HPTLC bioautography method was used to detect the presence of phenolic and antioxidant substances in SMCM seed extract (Figure 1), as this method provides rapid detection and localization of the active compounds in a plant extract. 

Chromatographic separation of the extract was achieved using dichloromethane/ethyl acetate (5:1) as eluent. Figure 1a, Figure 1b show the profiles of the separated spots after spraying with 90:10 methanol/sulphuric acid reagent after visualization under UV (366 nm) and visible light, respectively. After the run, the plates were further examined for DPPH active spots. Three pale-yellow colour spots on purple background were observed, thus indicating the presence of antioxidant substances (Figure 1c). In a separate HPTLC run, plates stained with Folin-Ciocalteu reagent demonstrated several phenolic active blue colour spots with yellow background (Figure 1d). Two phenolic and one unknown active spots exhibited antioxidant activity. This study shows the antioxidant activity was probably attributed to phenolic substances observed plates stained with Folin-Ciocalteu reagent.

## 3. Experimental

### 3.1. Chemicals

2,2-Diphenyl-1-picrylhydrazyl (DPPH), ferric chloride (FeCl_3_), TPTZ, xanthine, xanthine oxidase, Folin-Ciocalteu reagent, aluminium chloride (AlCl_3_), allopurinol, gallic acid, ascorbic acid, butylated hydroxytoluene (BHT) and catechin standards were obtained from Sigma-Aldrich (St Louis, MO, United States). HPLC grade methanol, dichloromethane, ethyl acetate, hydrogen peroxide, 95 % ethanol, hydrochloric acid (HCl), potassium ferricyaniade [K_3_Fe(CN)_6_] and trichloroacetic acid (TCA) were obtained from Merck (Germany). Ferric sulphate [Fe(SO_4_)_2_], sodium nitrate (NaNO_2_), and sodium hydroxide (NaOH), were purchased from Fluka (United States). Glass silica gel sheets were obtained from Merck.

### 3.2. Plant Materials

The *Swietenia mahagoni* seeds were collected in the state of Penang, Malaysia. The seeds were washed and cut into small pieces and dried for a week at 40 °C to remove the moisture content. The seeds were powdered using a blender (New Deluhe, Suruchi, India).

### 3.3. Sample Preparation

The powdered seeds (100 g) were extracted with methanol (400 ml) by the maceration method. The extract was filtered through filter paper (Whatman No.1), collected and concentrated in a rotary evaporator (RII0 Buchi,) in vacuum at 40 °C. The concentrated extract was dried to a consistent weight in an oven at 40 °C for three days prior to storage at -20 °C.

### 3.4. Xanthine Oxidase Inhibition Assay

Xanthine oxidase activity was determined by measuring the formation of uric acid from xanthine. SMCM seed extract at 1 mg/mL was prepared in 50 mM phosphate buffer solution (pH 7.0). Two millilitres of the sample was mixed with solution containing xanthine oxidase (2 mL, 0.4 U/mL) and xanthine (100 µM). After incubating at room temperature (24 ^o^C) for 3 minutes, uric acid production was determined by measuring the absorbance at 295 nm. The blank used was buffer and the control was a solution containing xanthine and xanthine oxidase. The inhibition percentage of xanthine oxidase activity was calculated according to the formula = (A_control_ –A_sample_) / A_control _x 100% [21].

### 3.5. Determination of Scavenging Activity against Hydrogen Peroxide

The SMCM seed extract radical scavenging activity against hydrogen peroxide was determined using the method of Ruch *et al.* [22]. Samples with different concentration were added to 0.1 M phosphate buffer solution (pH 7.4, 3.4 mL), respectively, and mixed with 43 mM hydrogen peroxide solution (0.6 mL). After 10 min, the reaction mixture absorbance was determined at 230 nm. The reaction mixture without sample was used as the blank. Ascorbic acid was used as a reference compound [9]. The percentage inhibition activity was calculated as: [(Abs. of control – Abs. of sample)/Abs. of control] × 100%.

### 3.6. Ferric-Reducing Antioxidant Power Assay (FRAP)

The FRAP assay was carried out according to the procedure of Benzine and Strain [10,11]. The FRAP reagent was prepared by mixing acetate buffer (25 mL, 300 mmol/L, pH 3.6), 10 mmol/L TPTZ solution (2.5 mL) in 40 mmol/L HCl and 20 mmol/L FeCl_3_ solution (2.5 mL) in proportions of 10:1:1 (v/v), respectively. The FRAP reagent was prepared fresh and warmed to 37 ^o^C in a water bath prior to use. One hundred and fifty microlitres of the sample was added to the FRAP reagent (4.5 mL). The absorbance of the reaction mixture was then recorded at 593 nm after 4 min; the assay was carried out in triplicates. The standard curve was constructed using FeSO_4_ solution (0.5-10 mg/mL). The results were expressed as µmol Fe (II)/g dry weight of plant material. L-ascorbic acid was also used as a comparative model for this assay.

*3.7. 2,2-Diphenyl-1-picrylhydrazyl (DPPH) Assay*

The free radical scavenging activity was measured by using DPPH assay. The quantitative estimation of radical scavenging activity was determined according to the methods described by Blois (1958) [23,5]. Five millilitres of 0.04% DPPH radical solution was added to SMCM seed extract solutions ranging from 0.031 to 2 mg/mL. The mixtures were vortex-mixed and kept under darkroom conditions for 30 min. The optical density (OD) was measured at 517 nm. Methanol was used as baseline control. Ascorbic acid, BHT and vitamin E were used as positive controls. The DPPH radical concentration was calculated using the following equation: Scavenging effect (%): (Ao – A_1_) × 100%/Ao, where Ao is the absorbance of the control reaction and A_1_ is the absorbance in the presence of the sample of the tested extracts. 

The IC_50_ (concentration providing 50% inhibition) was calculated graphically using a calibration curve in the linear range by plotting the extract concentration vs the corresponding scavenging effect. The antioxidant capacity was expressed as the antioxidant activity index (AAI), calculated as follow: AAI = final concentration of DPPH (mg·mL^-1^)/IC_50 _(mg·mL^-1^). Thus, the AAI was calculated after taking into consideration the mass of the DPPH and the mass of the tested extract in the reaction. The AAI of the tested extract is classified as poor antioxidant activity when AAI < 0.5, moderate antioxidant activity when the AAI is between 0.5 and 1.0, strong antioxidant when the AAI between is 1.0 and 2.0, and very strong when the AAI > 2.0 [13].

### 3.8. Determination of Phenolic and Flavonoids Content

The total phenolic content in the methanol extracts was measured using Folin-Ciocalteu reagent method [24]. The SMCM seed extract methanol solution (0.4 mL, 1mg/mL) was transferred into a test tube. To this solution, distilled water (1.0 mL) and Folin-Ciocalteu reagent (1.0 mL) were added, and the tubes shaken thoroughly. After 1 min, sodium carbonate solution (Na_2_CO_3_, 1.6 mL, 7.5%) was added and the mixture was allowed to stand for 30 min with intermittent shaking. A linear dose-response regression curve was generated using absorbance reading of gallic acid at the wavelength of 765 nm using UV-spectrophotometer (UV-160A, Shimadzu). The total phenolic compounds concentration in the extract was expressed as milligrams of gallic acid equivalent per gram of dry weight (mg GAE/g) of extract. The content of phenolic compounds in the plant extracts was calculated using this formula: C = A/B; where C is expressed as mg GAE/g dry weight of the extract; A is the equivalent concentration of gallic acid established from calibration curve (mg); and B is the dry weight of the extract (g) 

The total flavonoid content of the extract was determined according to colorimetric method as described in [25]. In brief, the sample solution (0.5 mL) was mixed with distilled water (2 mL) and subsequently with 5% NaNO_2_ solution (0.15 mL). After 6 min of incubation, 10% AlCl_3_ solution (0.15 mL) was added and then allowed to stand for 6 min, followed by additon of 4% NaOH solution (2 mL) to the mixture. Consequently, water was added to the sample to bring the final volume to 5 mL and the mixture was thoroughly mixed and allowed to stand for another 15 min. The mixture’s absorbance was determined at 510 nm. The total flavonoid content was expressed in mg of catechin per gram of extract.

### 3.9. High Performance Thin Layer Chromatography (HPTLC) study of Phenol and DPPH

High performance thin-layer chromatography (HPTLC) was performed on a silica gel glass plate (10 × 20 cm, Silica gel 60 F_254_, Merck). One gram powdered seed of *Swietenia mahogani* was extracted in methanol (5 mL) on rotary shaker (100 rpm) for 24 h [26]. The concentrated filtrate was used for chromatography separation in the HPTLC run. The extract was dissolved in ethyl acetate (10,000 ppm) and directly deposited on glass silica gel sheet using a Linomat 5 HPTLC extract loading machine (CAMAG, Switzerland). TLC plates were developed in a sandwich TLC chamber with dichloromethane/Ethyl acetate (5:1) solvent mixture as mobile solvent. After the run the plates were stained with Folin-Ciocalteu’s reagent and heated at 80^o^C/10min. The spots with antioxidant activity were determined using DPPH reagent. The glass silica gel sheets were dried and sprayed with 0.2% solution of DPPH in methanol. After 30 min, the antioxidant activity spots appear as pale-yellow spots on purple background [27].

### 3.10. Statistical Analysis

The data was presented as mean ± standard deviation (SD) for the three determinations.

## 4. Conclusions

SMCM seed methanol extract antioxidant capacity is comparable to that of other local Malaysian tropical fruits, herbs, edible seeds and nuts. Various assays were used to prove the antioxidant activity of the extract in this study. This is important since some plant extract with good antioxidant activity don’t necessarily show good antioxidant activity with any one particular one assay. Hence, more assays are needed to conclusively prove antioxidant activity. The presence on antioxidant bioactive compound(s) in the SMCM seed extract could possibly contribute to the antioxidant activity. The isolation of the bioactive antioxidant substances from SMCM seed extract warrrants further work.
